# The influence of flow velocity on the response of rheophilic fish to visual cues

**DOI:** 10.1371/journal.pone.0281741

**Published:** 2023-03-13

**Authors:** James Miles, Andrew S. Vowles, Paul S. Kemp

**Affiliations:** The International Centre for Ecohydraulics Research, Faculty of Engineering and Physical Sciences, Boldrewood Innovation Campus, University of Southampton, Southampton, United Kingdom; University of Florida, UNITED STATES

## Abstract

The strong association with visual cues exhibited by fish that prefer to inhabit flowing water (rheophilic species) may help reduce the energetic costs of maintaining position due to the provision of spatial points of reference. If this “Station Holding Hypothesis” is true, a positive relationship between the association with visual cues and flow velocity is expected. This hypothesis was tested experimentally by quantifying the response of common minnow (*Phoxinus phoxinus*) and brown trout (*Salmo trutta*) to visual cues under three flow velocities. In contradiction to the prediction, there was no evidence that the association with strong visual cues was positively related to flow velocity when fish were presented with vertical black stripes in an open channel flume, although interspecific variation in response was observed. The association with visual cues was relatively weak in trout, compared to minnow that spent 660% more time associated with the zone in which visual cues were present during the treatment, than the control when visual cues were absent. Trout tended to be more exploratory and made short visits to the area where visual cues were present, whereas minnow associated with the cues for longer. The strong association with visual cues independent of flow velocity exhibited by minnow and the weak association across all velocities by trout suggest that this behaviour is unlikely to reflect a strategy to minimise the energetic cost of maintaining position in flowing water. Minnow may have used the visual cues as a proxy indicator of physical structure that provides alternative benefits, such as refuge from predators. Trout may have employed alternative cues (e.g. mechanosensory) to seek more energetically favourable regions of the experimental area, reducing the importance of stationary visual stimuli.

## 1. Introduction

In face of the risk of being displaced by currents, many species of river-dwelling fish have adapted behaviours to orient into the flow (termed positive rheotaxis) and control their position [1]. This enables directional migration and holding station to intercept prey or detect odours within the current [2, 3]. Fish use mechanosensory and visual cues to control position relative to their surroundings [4], and considering that they perform rheotaxis with either vision or the lateral line inhibited, the relative role of each is still debated [5, 6]. The lateral line enables directionality of flow to be determined allowing fish to orient accordingly, but without a fixed point of reference it is unclear how they maintain position or monitor their relative motion within a flow [1]. Therefore, it is accepted that vision must play some role in station holding behaviour [7].

Fish control their speed and movement in dynamic flowing environments by stabilising their field of view using an innate optomotor response [8, 9], possibly as a position stabilising reflex to aid navigation and maintain cohesiveness in shoals [3, 10]. In addition to the optomotor response, fish tend to associate with strong visual reference points when navigating through both still [11, 12] and flowing water [12]. When offered a choice between a black and white striped and plain white wall, minnow almost always chose to associate with the wall displaying the strong visual cues (the stripes) [12]. There are currently two explanations for this behaviour. The first is that fish may associate with visual cues because they act as a proxy for physical structure in which to seek shelter from predators (the “Predator Refuge Hypothesis”). However, as the observed association is stronger in flowing water [12], a second hypothesis is that static visual cues provide a reliable point of reference from which to control position and swimming speed, and potentially reduce energetic costs of readjusting position (the “Station Holding Hypothesis”). Further work is needed to test these hypotheses.

Fish are known to associate with physical structure in flowing water to reduce energy expenditure. For example, they perform specialised behaviours, such as entraining, bow riding [13] and Kármán gaiting [14] to reduce energetic costs of maintaining position in complex flows. These behaviours take advantage of predictable hydrodynamic features, such as vortices produced by physical objects (e.g. cylinders or boulders), and vision is likely to play an important role when associating with fixed structures. For some rheophilic fishes, efficient station maintenance is critical to their behavioural ecology as it allows them to minimise energy expenditure, e.g. while intercepting passing invertebrate drift [15]. Association with visual cues might provide a means to help fish do this, either because it indicates the presence of physical structure and associated benefits, or because it provides information on spatial position. It is unknown, however, whether the presence of visual cues alone is sufficient to enhance swimming performance associated with station maintenance in the absence of the beneficial hydrodynamic characteristics produced by stationary physical objects within the flow.

In addition to ascertaining whether static visual cues provide fish energetic benefits in flowing water, resulting in a positive relationship between association with visual cues and flow velocity, a secondary question is how universal such a relationship might be. Stream-dwelling fish exhibit a diverse array of behavioural strategies linked to their ecology and life-history characteristics. For example, direct visual cues from external abiotic sources may be more important to species that exhibit solitary behaviours than those that live in groups and are thus able to obtain information from their conspecifics in addition to that provided from the surrounding environment. For example, red nose tetra (*Hemigrammus bleheri)* and minnow use visual information shared between conspecifics in flowing water to adopt group formations that optimise energy expenditure [16, 17]. Indeed, pairs of minnow enhance visual two-way information transfer in flow by adopting positions side-by-side compared to a tandem formation (follow-the-leader) in still water in which information transfer is one-way [17]. However, whether fish can gain similar benefits by aligning alongside stationary environmental visual cues is unexplored.

In this study we adopted a reductionist approach by using single fish only in each trial to control for the confounding effect of visual information transfer between group members, and used two common stream-dwelling rheophilic species, minnow and brown trout, with different life-history characteristics to quantify interspecific variation. We explored: (1) the relationship between association with visual cues and flow velocity, a proxy for energy expenditure; and (2) interspecific variation in response exhibited. Two separate experiments, one per species, were conducted at three flow velocities to confirm that the subject fish (a) associate with visual cues and to test the prediction that (b) there is a positive relationship between association with visual cues and flow velocity (supporting the “Station Holding Hypothesis”). Furthermore, we tested the prediction that (c) interspecific differences in response will be observed, with typically shoal-dwelling minnow exhibiting a weaker association with visual cues than more solitary trout because they are better adapted to obtain information from alternative sources (e.g. social transfer of information between group members). Conversely, we assumed that trout are likely adapted to rely on external static visual cues to enable them to better hold station in the water column against the flow, e.g. to feed on passing particles of invertebrate drift.

## 2. Method

Experiments were designed to quantify the behaviour of minnow and trout in the presence and absence (control) of visual cues at three flow velocities (low, mid and high), resulting in a total of six treatments. Treatment flow velocities were adjusted to accommodate interspecific differences in swimming capabilities. Between 10 and 21 replicates were conducted for each treatment, resulting in a total of 109 and 76 trials using individual minnow and trout, respectively.

### 2.1 Experimental setup

**Fig 1:**
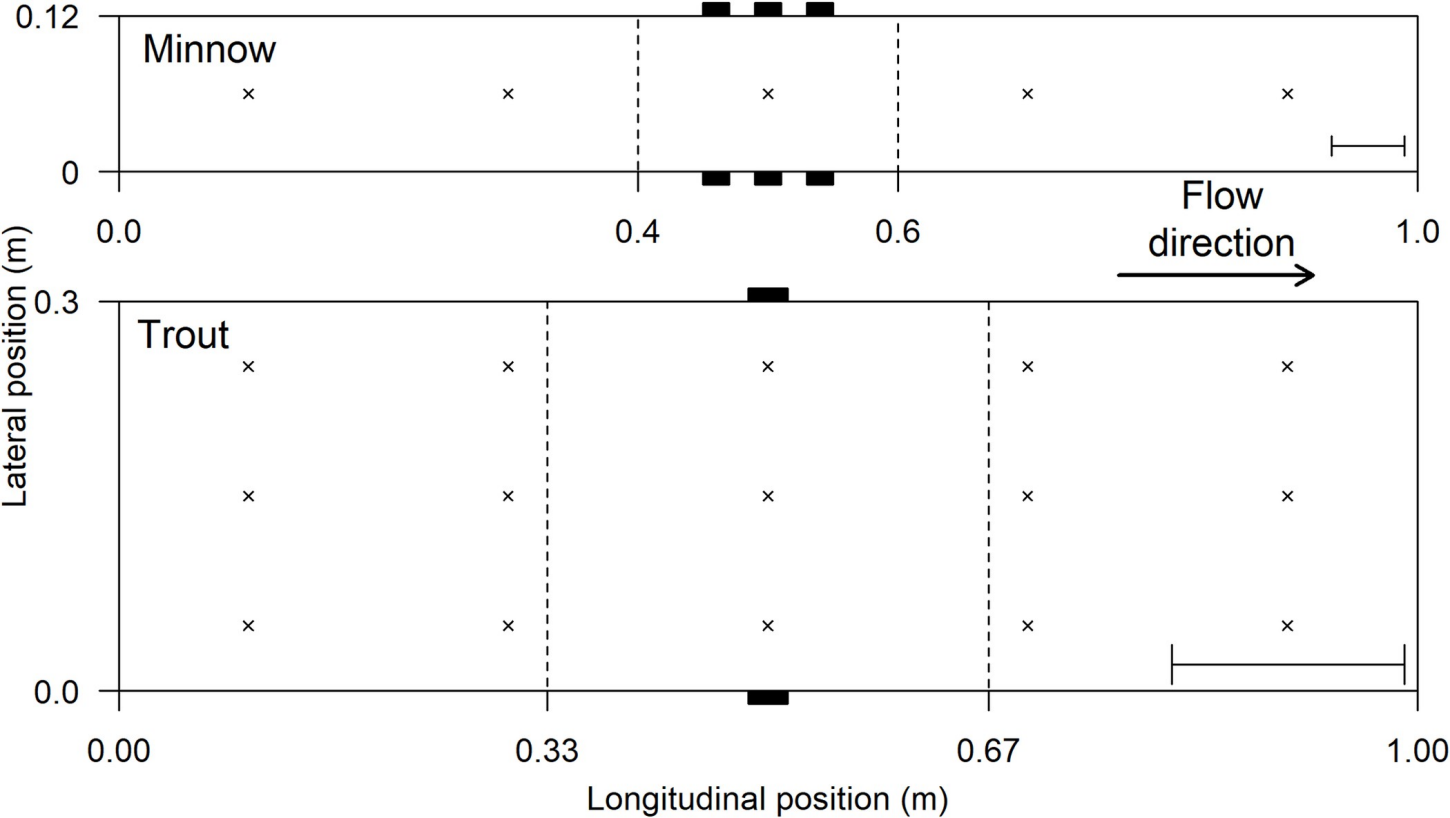
Plan view of the experimental set-up for minnow (top) and trout (bottom). The experimental area was isolated from the rest of the flume using tubular flow straighteners (upstream and downstream). Dotted lines display the central zone and small black boxes at the sides of the flume the position of the visual cues (vertical stripes). The positions of flow measurements are indicated by small crosses. Flow was from left to right. The scale bar in the bottom left of each box represents the mean fish fork length for that species.

Two experiments were conducted at the International Centre for Ecohydraulics Research (ICER; University of Southampton, UK) facility using a rectangular, open-channel recirculating flume (12.0 m x 0.3 m x 0.4 m) between 1 and 24 March 2021 (minnow) and 13 and 20 January 2020 (trout). The experimental area (1.0 m length) was separated from the remainder of the flume by flow straightening screens (consisting of 100 mm long and 7 mm diameter polycarbonate tubes, Tubus Bauer) that also minimised turbulence (Fig 1). Flume width differed between experiments (0.12 m for minnow and 0.3 m for trout) to accommodate interspecific differences is body size. Within the experimental area, white laminate PVC sheeting was secured to the base and walls to minimise visual reference points and maximise contrast to improve video processing. Any reference points outside the flume were blocked using a blackout hide, within which indirect, diffused LED strip lights (Brillihood—LED-Batten-4FT-36 W, 2950 lumen, frequency peaks: 450 nm & 550–600 nm) provided uniform illumination.

Visual cues were provided by vertical black and white stripes on the centre of each wall. Three 20 mm wide black stripes and a single 35 mm black stripe was used for minnow and trout, respectively. Stripe width differed between treatments in-line with flume width and guaranteed the cues fell within the optimum visual acuity range of the test species (measured from centre of flume: minnow = 0.03 cycles/degree; trout = 0.04 cycles/degree) [18, 19]. Three stripes were used for minnow to improve visual cue detection in the narrower channel. During the controls, both walls were devoid of strong visual cues (stripes). A Logitech webcam (HD Pro Webcam C920; 30 frames/second, Resolution = 1080p) positioned 90 cm above the centre of the experimental area captured fish movement and behaviour.

### 2.2 Flow velocities

The three flow velocities (low, mid and high; Table 1) for minnow equated to approximately 1.5, 2.5, and 3.5 body lengths per second (BLs^-1^). This is within the sustained swimming speed measured in an open channel for this species [20]. For trout, the velocities were approximately 1, 2 and 3 BLs^-1^ and also within their sustained swimming speed [21]. Unidirectional flow velocity was measured at five equidistant points (20 cm apart) along the midline of the channel at 50% water depth using a Nortek Vectrino+ 16 MHz Acoustic Doppler Velocimeter (ADV) at a sampling frequency of 50 Hz for 30 seconds in each location. As the experimental area was wider for trout than for minnow, three lateral measurements were also taken, totalling 15 locations (Fig 1). Only measurements with a signal-to-noise ratio (SNR) > 20 dB and a correlation > 80% were used to estimate flow velocity. Water depth was maintained at 10 cm (minnow) and 12 cm (trout) for all velocity regimes.

**Table 1:** Summary of mean unidirectional flow velocities (± SD) and sample sizes for low, medium and high velocity treatments for common minnow (*Phoxinus phoxinus*) and brown trout (*Salmo trutta*).

Species	Velocity	Velocity (cms^-1^)	SD	Sample size (N)	
				Control	Treatment
Common minnow	low	9.0	± 1.2	16	21
	mid	15.0	± 1.7	15	20
	high	19.3	± 1.5	16	21
Brown trout	low	17.5	± 0.2	10	14
	mid	33.3	± 0.3	10	15
	high	45.4	± 0.2	10	14

### 2.3 Capture and maintenance of experimental fish

Minnow (n = 109; mean fork length ± standard deviation (SD) = 54 ± 6 mm; mean weight ± SD = 2.07 ± 0.74 g) were collected from the River Itchen (Riverside Park, Southampton, UK, lat: 50°56’05.2"N long: 1°22’23.9"W) on 25 February and 5 March 2021 using a 5 m seine net. They were transported to holding tanks at the University of Southampton in 50 l containers of aerated river water. Minnow were acclimated in a 200 l perforated, aerated container within the flume sump as the water temperature could be controlled to closely match that of the source river (11°C). Water temperature was slowly increased over three days to the laboratory ambient (15°C). Prior to use in the trials, minnow were moved to four 120 l holding tanks (mean temperature = 15.8 ± 0.6°C; maximum stocking density = 0.59 kg m^-3^) for a minimum of 72 hours. Trout (n = 76; mean fork length = 179 ± 9.9 mm; mean weight = 76.9 ± 11.4 g) were collected from a nearby trout farm and transported to the University of Southampton in a 250 l aerated tank on 10 January 2020. Fish were held in a 1200 l temperature-controlled holding tank (mean temperature = 10.9 ± 0.7°C; maximum stocking density = 5.84 kg m^-3^) for 72 hours prior to use in trials. Feeding and water quality testing was performed daily for both species and water regularly changed (50%) to ensure high quality was maintained (ammonia < 0.25 mg l^−1^, nitrite < 0.25 mg l^−1^, and nitrate < 50 mg l^−1^). Lighting regimes matched the natural photoperiod throughout the study for both minnow and trout.

### 2.4 Experimental protocol

Minnow and trout were acclimated to the flume water outside of the experimental area. This enabled fish to acclimate while other trials were conducted. Minnows were isolated and acclimated to the water temperature and illumination for at least 15 minutes prior to being released into the experimental area. Trout were acclimated to the flume water over night within the flume sump and an individual was isolated from the rest of the group for at least 30 minutes prior to being released into the experimental area. An average of seven and 9.5 trials were performed each day for minnow and trout, respectively.

Film recording commenced when fish were released into and allowed to volitionally explore the experimental area. Trials lasted for 30 minutes after which the fish were removed from the flume before being measured (fork length, mm) and weighed (g). Fish length (ANOVA: Minnow: F_3,104_ = 0.76, p = 0.51; Trout: F_3,72_ = 0.31, p = 0.82) and weight (ANOVA: Minnow: F_3,104_ = 0.62, p = 0.61; Trout: F_3,73_ = 0.71, p = 0.55) did not differ between treatments. Treatments were pseudo-randomised to minimise potential effects of confounding variables. The mean ± SD flume water temperature did not differ between treatments throughout the experimental period (ANOVA: Minnow: 15.0 ± 0.6°C; F_3,105_ = 0.94, p = 0.43; Trout: 12.6 ± 0.31°C; F_3,73_ = 0.74, p = 0.48).

### 2.5 Behavioural metrics

Video data was processed in Matlab [22] using an automated custom written fish-tracking function that recorded the coordinates of the fish’s head every third of a second for the entire 30-minute trial by measuring changes in contrast between the fish and the background. The position of the fish was taken from the anterior end of the fish’s silhouette, which was deemed to be the approximate location of the eye and therefore the most appropriate measure of position relative to the visual cues. The first five minutes of each trial were excluded from analysis to provide time for fish to acclimate to their surroundings and exclude any erratic behaviour associated with introduction to a new environment. No startle responses or escape behaviours were observed after this period. Using the coordinates recorded, three metrics were calculated to analyse the behaviour of fish relative to the central (test) zone between treatments: (1) *Association*, (2) *Number of visits*, and (3) *Visit duration* (Table 2). Fish located in the central zone were deemed to associate with visual cues under the treatments (Fig 1). The same metrics were used for both species.

**Table 2:** Definitions of the behavioural metrics devised to statistically analyse fish behaviour during each trial.

Behavioural metric	Definition	Transformation	
		Minnow	Trout
*Association*	Percentage of trial time spent within the central zone (%)	Square-root	Natural log
*Number of visits*	Number of visits to the central zone from either upstream or downstream end of the experimental area	Square-root	Square-root
*Visit duration*	The time spent in the central zone on any one occasion (s)	Natural log	Natural log

### 2.6 Statistical analysis

Linear models were constructed in R [23] and used to assess all metrics. Shapiro-Wilk and Levene’s tests were performed to check for normality and homogeneity of variance, respectively. Where appropriate natural log or square-root transformations were performed to normalise the response variables (Table 2). The behavioural metrics were used as response variables with visual cue and flow velocity as explanatory variables. Interactions between explanatory variables were assessed for both species. Chi-square and F statistics were calculated using the car package [24], and post-hoc tests using the phia package for analysis of interaction terms [25].

### 2.7 Animal welfare

Ethics was reviewed by the Animal Welfare and Ethics Review Board and approval granted by the University of Southampton Ethics and Research Governance committee (ID: 52245). Permission was obtained from the UK Environment Agency to source common minnows from their natural habitat and to return them shortly after completion of the trials. Individuals were handled with care, and handling time was kept to a minimum. There was no evidence of stress or fatigue from exposure to the visual cues or flowing water treatments during the 30-minute trial time.

## 3. Results

### 3.1 Association with the central zone

**Fig 2:**
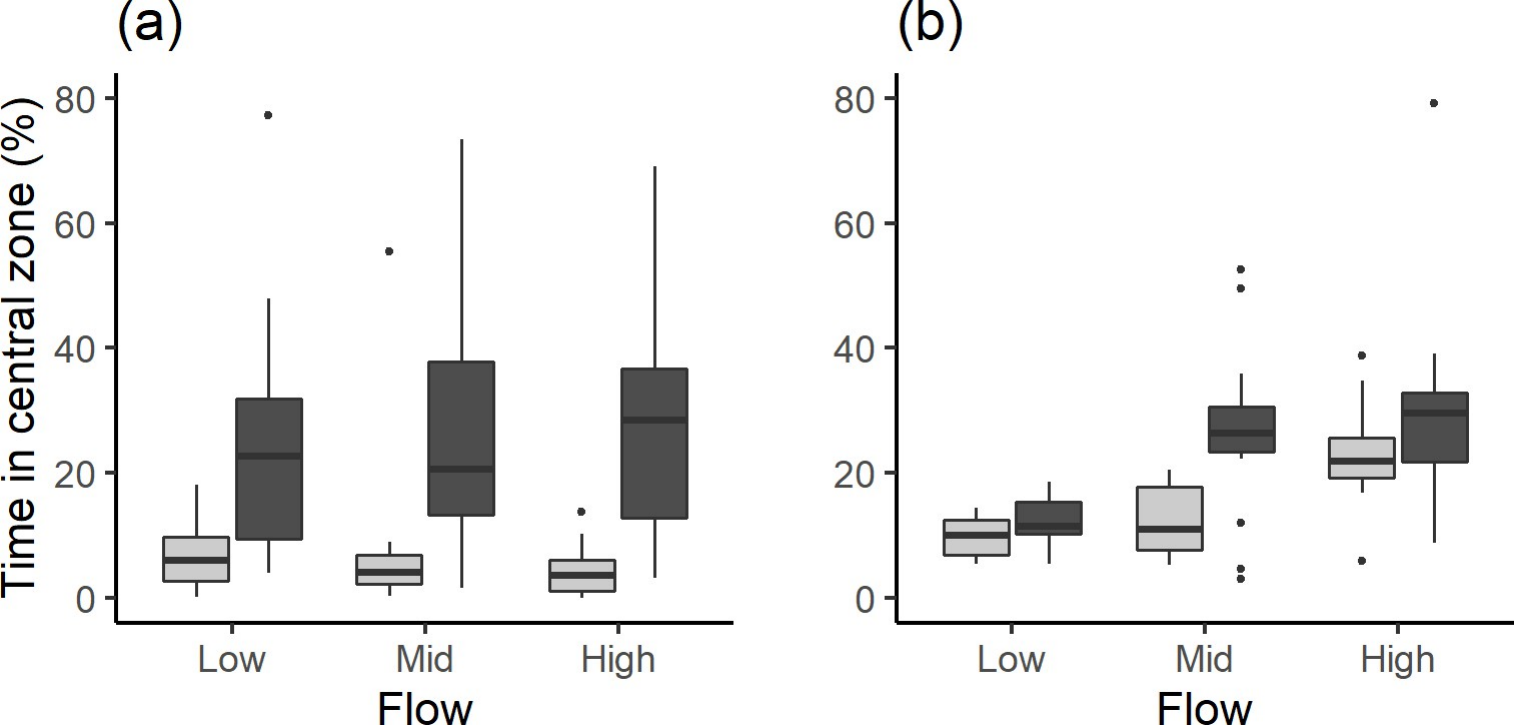
The percentage of time spent in the central zone of a flume with (dark grey) and without visual cues (light grey–the control) under three flow velocities for (a) common minnow and (b) brown trout.

Minnow associated with the visual cues, spending 6.6 times longer occupying the central zone during the treatment (median [IQR]: 28.5 [31.7]% of trial time) compared to the control, when visual cues were absent (4.3 [6.7]%; ANOVA: F_1,101_ = 72.4, p < 0.001; R-squared = 0.428; Fig 2A). However, the proportion of time associated with visual cues was not related to flow velocity (ANOVA: F_2,101_ = 1.33, p = 0.27).

For trout, we expected that the proportion of time spent occupying the central zone would remain constant in the absence of visual cues and increase with flow velocity when they are present. However, there was no interaction between visual cues and flow velocity (ANOVA: F_(2,67)_ = 1.07, p = 0.35) despite the fact that trout spent a greater proportion of time in the central zone when visual cues (ANOVA: F_(1, 67)_ = 7.7, p = 0.007; R-squared = 0.085) were present and at higher flow velocities (ANOVA: F_(2, 67)_ = 11.7, p < 0.001). As such, flow velocity did not influence the proportion of time trout spent associating with visual cues (Fig 2B). Overall, trout spent roughly 45% more time in the central zone when visual cues were present (median [IQR] = 20.5 [15]% of time) compared to the control (median [IQR] = 14.0 [11.3]%), and 23.9 [11.6]% at the highest flow velocity compared to 11.5 [6.7]% at the lowest (Fig 2B). *Association* was greater at the high compared to mid (F_1,67_ = 6.9, p = 0.02) and low flow velocity (ANOVA: F_1,67_ = 22.6, p < 0.001), and *Association* at the mid velocity was greater than low velocity (ANOVA: F_1,67_ = 4.7, p = 0.04).

### 3.2 Number of visits to the central zone

**Fig 3:**
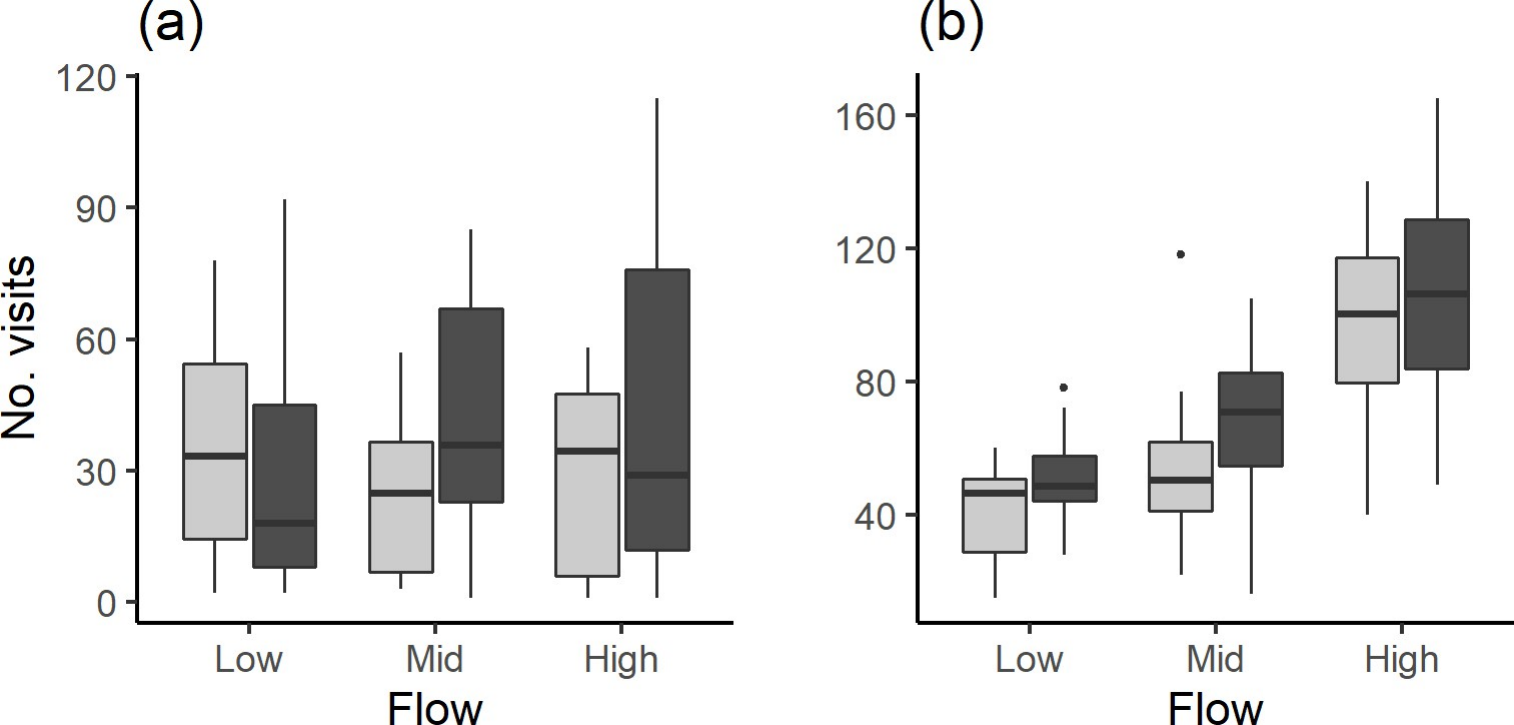
The *Number of visits* to the central zone for the control (light grey) and visual cue treatment (dark grey) at the three flow velocities for (a) common minnow and (b) brown trout. Note the difference in y-axis scale.

For minnow, neither the presence of visual cues (ANOVA: F_1,101_ = 1.32, p = 0.25) nor flow velocity (ANOVA: F_2,101_ = 0.15; p = 0.86) affected the *Number of visits* to the central zone (Fig 3A).

For trout, velocity influenced the *Number of visits* to the central zone (ANOVA: F_2,69_ = 27.5, p < 0.001) but the presence of visual cues did not (ANOVA: F_1,67_ = 2.33, p = 0.13). There was no interaction between fixed factors (ANOVA: F_2,67_ = 0.03, p = 0.97). Overall trout made over twice as many visits to the central zone at the highest (median [IQR] = 114 [57.2]) compared to the lowest velocity (median [IQR] = 54.5 [30.2]; Fig 3B). Post hoc tests indicated a greater *Number of visits* to the central zone at the high compared to the mid (F_1,67_ = 25.9, p = < 0.001) and low flow velocities (ANOVA: F_1,67_ = 50.1, p = < 0.001) and at the mid compared to the low flow velocity (ANOVA: F_1,67_ = 4.2, p = 0.045).

### 3.3 Duration of visits to central zone

**Fig 4:**
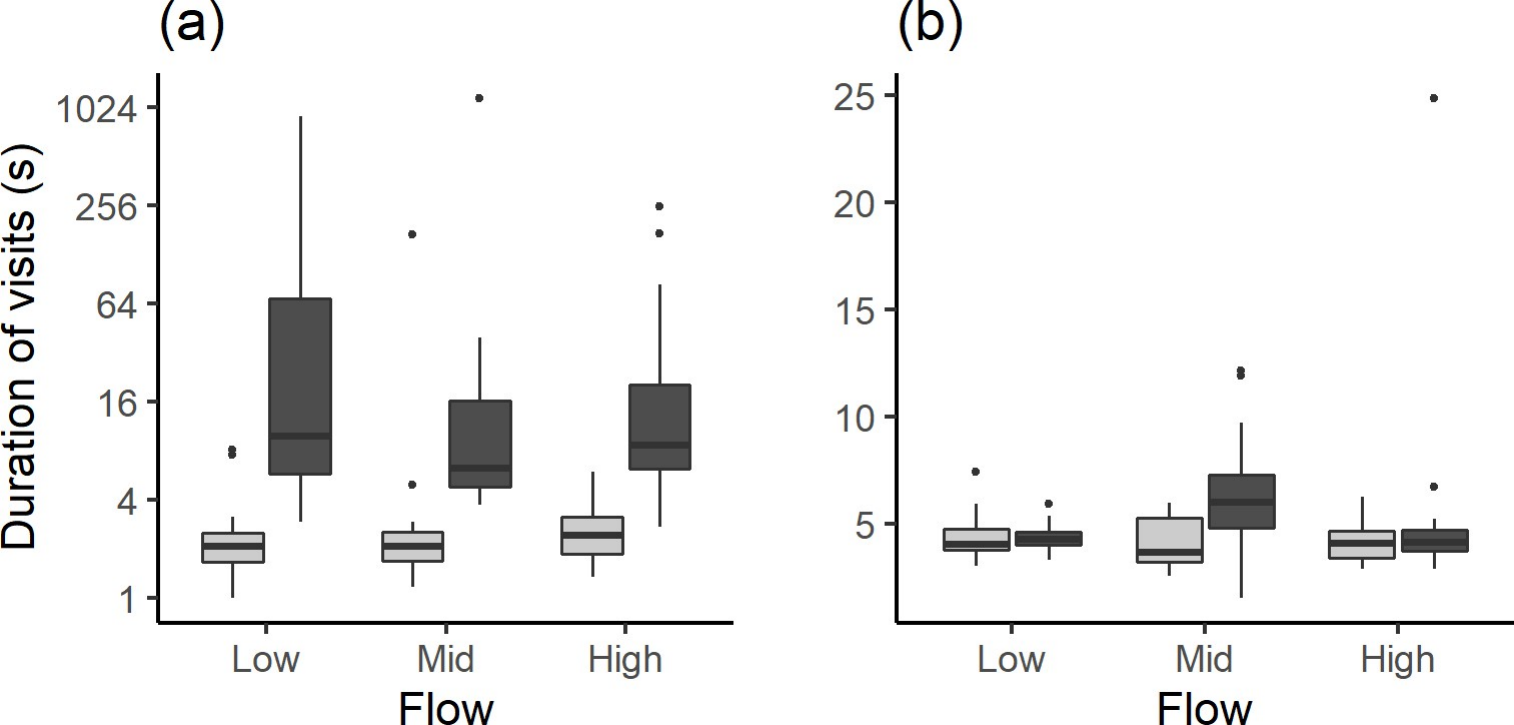
Median *Duration of visits* to the central zone for the control (light grey) and visual cue treatment (dark grey) at the three flow velocities for (a) common minnow and (b) brown trout. Note the difference in y-axis scale—the scale for minnow has been log transformed.

For minnow, the median [IQR] *Duration of visits* to the central zone was approximately three times longer when visual cues were present (6.74 [7.12] s) compared to the control (2.10 [1.11] s; ANOVA: F_(1,89)_ = 107.3, p < 0.001). There was no difference in *Duration of visits* between the three flow velocities (ANOVA: F_2,89_ = 0.77, p = 0.46; Fig 4A) and there was no interaction between explanatory variables (ANOVA: F_2,89_ = 0.81, p = 0.45).

For trout, the *Duration of visits* to the central zone did not differ with visual cue (ANOVA: F_1,66_ = 2.72, p = 0.10) or flow velocity (ANOVA: F_2,66_ = 2.61, p = 0.08; Fig 4B).

## 4. Discussion

In this study we proposed and tested the “Station Holding Hypothesis” premised on previous observations that stream-dwelling fish tend to associate with static visual cues [11, 12]. The proposition is that, in a flowing environment, stationary visual cues provide spatial points of reference that enable fish to remain in a fixed place, thus limiting costs of displacement, such as the need to continuously re-evaluate and adjust orientation to maintain position in a suitable location (e.g. to feed on invertebrate drift or remain unseen by a predator). By adopting an experimental approach, we first confirmed the findings of previous studies that rheophilic fish, in this case the common minnow and brown trout, associate with static visual cues. Second, we tested the prediction that the association with static visual cues is positively related to flow velocity, assuming the “Station Holding Hypothesis” to be true. We found that the association with visual cues was not influenced by flow velocity in either species, contradicting the “Station Holding Hypothesis”. The responses displayed suggest that visual cues are not primarily used to control position for energetic benefit, at least under the conditions created in this experiment, and that the observed association is due to some other underlying mechanism. Finally, we also explored interspecific differences in response to visual cues. Minnow showed a relatively high degree of affinity to the vertical stripes, exemplified by the consistently higher time spent in the central zone when visual cues were present across all flow velocities compared with the control. Conversely, the association specifically with visual cues was comparatively weaker for trout, only exhibiting a higher degree of association compared with the control at the mid flow velocity. This is also evident in the higher effect size (% difference) for association in the central zone for minnow compared with trout. This may be due to the high levels of exploratory behaviour under the fastest velocity condition experienced, even though they are well adapted to holding position in flowing water [26].

The lack of a relationship between the association with static visual cues and flow velocity in either species was unexpected, resulting in the rejection of the “Station Holding Hypothesis”. An alternative explanation may be that, rather than gaining energetic benefits through more efficient station maintenance against the flow, the vertical stripes might provide a proxy indicator of physical structure or an opportunity for crypsis. This alternative “Predator Refuge Hypothesis” may explain an association with the visual cues in three flow velocity regimes, and the previously observed strong association with visual cues in static water where costs of station holding are irrelevant [12]. Indeed, physically complex habitats, such as patches of submerged vegetation, are utilised by fish to reduce predatory encounters [27]. In southern English chalk streams, trout density is positively related with water depth in winter and spring when availability of instream macrophyte cover declines [28]. This behaviour appears to reflect a predator avoidance (risk minimising) strategy similar to that described for trout in northern boreal river systems where diurnal activity and habitat use change seasonally to minimise fitness costs in response to harsh winter conditions that may elevate predation risk [29]. For minnow, more time is spent associated with physical refuge following a simulated predation attempt when compared with three-spined stickleback (*Gasterosteus aculeatus*) [30]. Behavioural defences, therefore, appear to be more strongly adopted by species that lack morphological defences (e.g. the dorsal and ventral spines of the stickleback), and this provides additional support that the “Predator Refuge Hypothesis” may have influenced the behaviours observed in this study. Association with visual cues as a proxy for cover may have been particularly important for fish in this study as the experimental design precluded the use of other behaviour defences, such as shoaling.

It is not immediately clear why minnow exhibited a relatively strong association with visual cues and trout did not, but considering minnow are predominantly social while trout may be more solitary and are adept at holding position in flowing water, the observed differences in their responses may reflect variation in behavioural ecology. Minnow are often found in large shoals [31], thus enabling individuals within the group to benefit from access to social information [32]. As such, we assumed that, compared to trout, minnow would be adapted to rely on information provided by other group members, in addition to that available from external visual sources, reducing the relative importance of the latter even when the minnows are solitary. In fact, we observed the opposite; minnow showed a greater association with visual cues than trout, potentially reflecting a need to modify behaviour in the absence of other group members. In this reductionist study, solitary minnow were used in each trial and thus socially transferred information was absent, potentially causing the fish to increase their dependency on external abiotic visual information. Indeed, others have reported shifts in minnow behaviour depending on social context, i.e. whether they are solitary or members of a group. For example, when groups of minnow were presented with an acoustic stimulus, they exhibited a consistent anti-predator response by becoming more polarised and cohesive, whereas individual fish responses were more chaotic, swimming at a faster rate with frequent changes of direction [33].

While stream-dwelling species of trout may form shoals and loose aggregations [34], they are also commonly observed to behave in a solitary manner, frequently holding station in the water column close to a structure (e.g. boulder, wood) or bank, as part of a drift feeding strategy and form of motion camouflage that employs background matching [35, 36]. We predicted that trout would associate with visual cues in flowing water in a manner consistent with the “Station Holding Hypothesis” because of the potential benefit of enhancing drift foraging efficiency at higher, more energetically costly flow velocities. Instead, trout displayed higher exploratory behaviours at the faster velocities rather than holding station. Of course, in this experiment we expected fish to follow a “rule of thumb” and display behaviours similar to those observed in the wild, even in the absence of food. However, such an expectation may be unrealistic and a shift from station holding to active exploration may simply reflect a lack of motivation to maintain feeding positions when food was absent. Furthermore, it is also important to recognise that the trout used in this study were sourced from a hatchery (minnow were captured from a river), potentially providing an alternative explanation for the observed differences in behaviour (see Braithwaite & Girvan, 2003for origin related differences in foraging and use of visual cues between river and pond populations of stickleback [37]).

In this reductionist study, we focused on the use of visual cues by fish, while intentionally ignoring the importance of other sensory modalities. However, station holding is a multisensory process [1], and the role of vision will only partially explain the ability of fish to maintain position against the flow. Fish are capable of making decisions based on multisensory information obtained simultaneously [38], and may preferentially utilise the information provided by those senses that are most reliable and robust relative to the spatial and temporal context / environment (e.g. mechanosensory and acoustic stimuli are likely to be more important than visual cues in turbid rivers or when dark) [38]. Rheophilic fish are known to use predictable hydrodynamic structures, such as Kármán vortices, to reduce locomotory costs [39]. Station holding trout in our experiment may have also used features of the hydrodynamic environment to support information provided by visual cues to fix on a specific point.

The influence of visual cues on the behaviour of both minnow and trout was evident as both species spent significantly more time in the central zone during the treatment. However, the consistent lack of correlation between association with visual cues and flow velocity contradicts the “Station Holding Hypothesis”. Future research should explore alternative explanations, such as the “Predator Refuge Hypothesis”.
